# Supplementary material for: MiR 208a Regulates Mitochondrial Biogenesis in Metabolically Challenged Cardiomyocytes
Source: Cells. 2021 Nov 13;10(11):3152. doi: 10.3390/cells10113152 (PMC8622724; doi:10.3390/cells10113152)
Supplement: Supplementary file 1 [file cells-10-03152-s001.zip › cells-1444174-supplementary.pdf]

SUPPLEMENTARY MATERIAL

Human 208 primers:

- Taqman hsa-miRNA 208 Primers (Cat No: 000511)
- Taqman hsa-U6 snRNA primers (Cat No: 001973)

Table S1. Primers for the miR 208a target mRNAs for human cardiomyocytes

	**Human target gene**	**Primers**	**Sequence**
1	**Stanniocalcin-1 (Stc1)**	**Forward**	**GGCGACCACCAAAGTCAAAC**
		**Reverse**	**TACTTGTCGCATTGGGGTCC**
2	**Nuclear Receptor Coactivator 7 (NCOA7)**	**Forward**	**GCCCTGGAAACTCCCTCTTC**
		**Reverse**	**AGCCCAACATCTAGGGGCTA**
3	**Mediator Complex Subunit 7 (MED)7**	**Forward**	**CCCTTTGGAAAGTCAGGGCA**
		**Reverse**	**TCTCAGCTGTTTCAAGCCGT**
4	**Mitochondrial Ribosomal Protein S28 (MRPS28)**	**Forward**	**ACAGATGGGACCTGCAAAGG**
		**Reverse**	**TCTAATAGCCGCAACCGGAC**

5	**Sortin Nexin 10 (SNX10)**	**Forward**	**AGTGTCTGGGTTCGAGATCCT**
		**Reverse**	**GGTAACACTGCCCCACTGAT**
6	**ATP synthase subunit 5, mitochondrial (ATP5)**	**Forward**	**TACTCGAAGCCTCGATTGGC**
		**Reverse**	**GCGGGCTAAACAGACGTGTA**
7	**Beta actin**	**Forward**	**CCTTTGCCGATCCGCCG**
		**Reverse**	**AACATGATCTGGGTCATCTTCTCGC**
8	**Mitochondrial Contact Site and Cristae Organizing System Protein 1 (MINOS1)**	**Forward**	**CGGATGCGGTCGTGAAGATA**
		**Reverse**	**ACTGCTCCTGCTCTTTGACA**

Table S2. List of antibodies used in this study. Ms: Mouse, Rb: Rabbit

**Antibodies**	**Source**	**Catalog number:**
Beta actin	Ms	Ab197277
GAPDH	Rb	2118S
Rodent Total OXPHOS cocktail	--	MS604/H4664
• NDUFS3	--	MS110/C1924
• Complex V beta	--	MS503
• Complex III	--	MS305

• Complex IV sub	--	MS407
• Complex II	--	Ab110410/D0604
Anti-OXPHOS complex IV	--	459600 (Invitrogen)
MHC-β	Ms	Sc376157
Citrate Synthase	Rb	Ab96600
CPT1B	Rb	Ab104662
Fibronectin	Rb	Ab23750
ANP	Rb	Ab91250
PGC1 alpha	Rb	Ab54481
MHC-α	Rb	Ab224046
Thyroid Hormone Receptor	Rb	Ab53729
ACAD	Rb	Ab196655
NDUFS4	Ms	Ab87399
TFAM	Rb	Ab138351
ACADM/MCAD	Rb	2758-1
GAPDH	Ms	Sc365062
NRF1	Rb	Ab34682
ANP	Ms	Sc515701
CPT1	Ms	Sc515577
Cytochrome C	Ms	ab13575

Figure S1A: Stanniocalcin 1

```
                                ....700...
5'   ...GUAUCUUUAACAAUUGUCUUAAA...   position 696-702 of STC1 3' UTR
           ||||        ||||||
3'    UGUUUGGAAAACGAG--CAGAAUA       hsa-miR-208a-3p
```

```
           ....700...
Human    UUGUCUUAAAA-C-CA
Chimp    UUGUCUUAAAA-C-CA
Rhesus   UUGUCUUAAAA-C-CA
Squirrel UUGUCUUAAAA
Mouse    UUGUCUUAAAA
Rat      UUGUCUUAAAA
Rabbit   UUGUCUUAAAA
```

```
                ..400.......410....
5'   ...UUUAUUUAAAUGCAUGUCUUAAA...   position 409-415 of STC1 3' UTR
          |||         ||||||
3'    UGUUCGAAAAACGAG--CAGAAUA       hsa-miR-208a-3p
```

```
..400.......410....
UUUAAAUGCAUGUCUUAA----A-UG
```

Figure S1B: MED7

```
          ....70 .......
Human    GACAGU-CUUAAGA
Chimp    GACAGU-CUUAAGA
Rhesus   GACAGU-CUUAAGA
Squirrel GACAGU-CUUAAAA
Mouse    GACAGU-CUUAA
Rat      GACAGU-CUUAA
Rabbit   GGCAGU-CUUAA
```

```
                    .....70.......
5'  ...AUUUUCUUUUGGACAGUCUUAAG...   Position 69-75 of MED7 3' UTR
           |||||      ||||||
3'     UGUUCGAAAAACGAGCAGAAUA        hsa-miR-208a-3p
```

Figure S1C: SNX10

```
                         ...190...
5'  ...GGUAAGCUUCUGUGU-AAAGCUAA..   position 188-194 of SNX10 3' UTR
           |||       ||||||
3'      UGUAUUAAGCUCGUUUUUCGAG      hsa-miR-208a-5p
```

```
                    .1260 ...1270
5'   ...UUUCACUAAGAGUUUAAAAGCUA..   position 1265-1272 of SNX10 3' UTR
                       |||||||
3'       CAUAUUGGGCCCGGUUUUCGAG     hsa-miR-208a-5p
```

```
                       ......1330..
5'  ...AGAACCAACAGAAAAAAAAGCUA..    position 1325-1332 of SNX10 3' UTR
                       |||||||
3'      UGUAUUAAGCUCGUUUUUCGAG      hsa-miR-208a-5p
```

Figure S1D: MRPS28

```
                    .......230.
5'    ...GAAGAUAUAAUAAAUAAAAGCUA...  Position 226-233 of MRPS28 3' UTR
                        |||||||
3'       CAUAUUGGGCCCGGUUUUCGAG      hsa-miR-208a-5p
```

Figure S1E: MINOS1

```
            190........200......
5'    ...AGUUGUAAGGAGGUGGUCUUAAA..   Position 200-206 of MINOS1 3' UTR
                        ||||||
3'       UGUUCGAAAAACGAGCAGAAUA      hsa-miR-208a-3p
```

```
          .........200.......
Human     UAAGGAGG-UGG--------UCU-U-----AAAUAAA----
Chimp     UAAGGAGG-UGG--------UCU-U-----AAAUAAA----
Rhesus    UAAGGAGG-UGG--------UCU-U-----AAAUAAA----
Squirrel  UAAGGAGG-UGA--------UCU-U-----AAAUAAA----
Mouse     UAAGGAGG------------UCU-U-----AAAUAAA----
Rat       UAAGGAGG------------UCU-U-----AAAUAAA----
Rabbit    UGAGGAGA-UGG--------UCU-UAAGAGAAAUAAA----
```

Supplementary Figure S1 (in support of Figure 2 in the main manuscript). mRNA targets of miR 208a in cardiomyocytes incubated in diabetogenic conditions. The alignment between miR 208a sequence and specific mRNAs as well as the level of conservation of those sequences in different species are also shown.

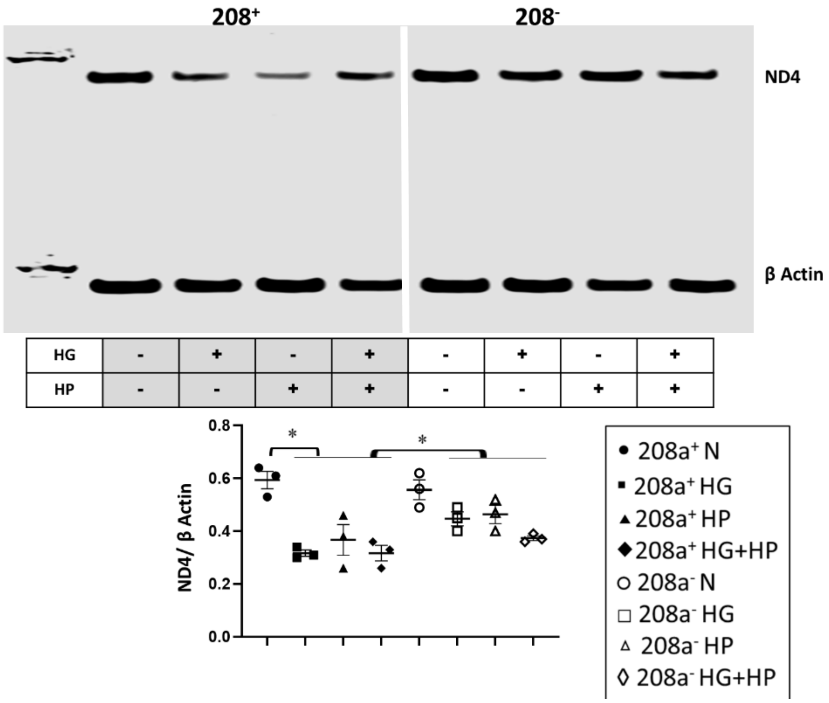

Supplementary Figure S2 (in support of Figure 4C in the main manuscript). SV40 human cardiomyocytes were incubated in DMEM with either 5 mM glucose, 25 mM glucose (high glucose, HG) or 20 μM bovine serum albumin (BSA)-bound palmitate (high palmitate, HP) for 24 hours. Mitochondrial DNA**.** Total DNA was subjected to quantitative real time PCR using a designed primer for mitochondrial NADH dehydrogenase 4 (ND4). The results are expressed as Mean ± SEM of three independent experiments. *P < 0.0

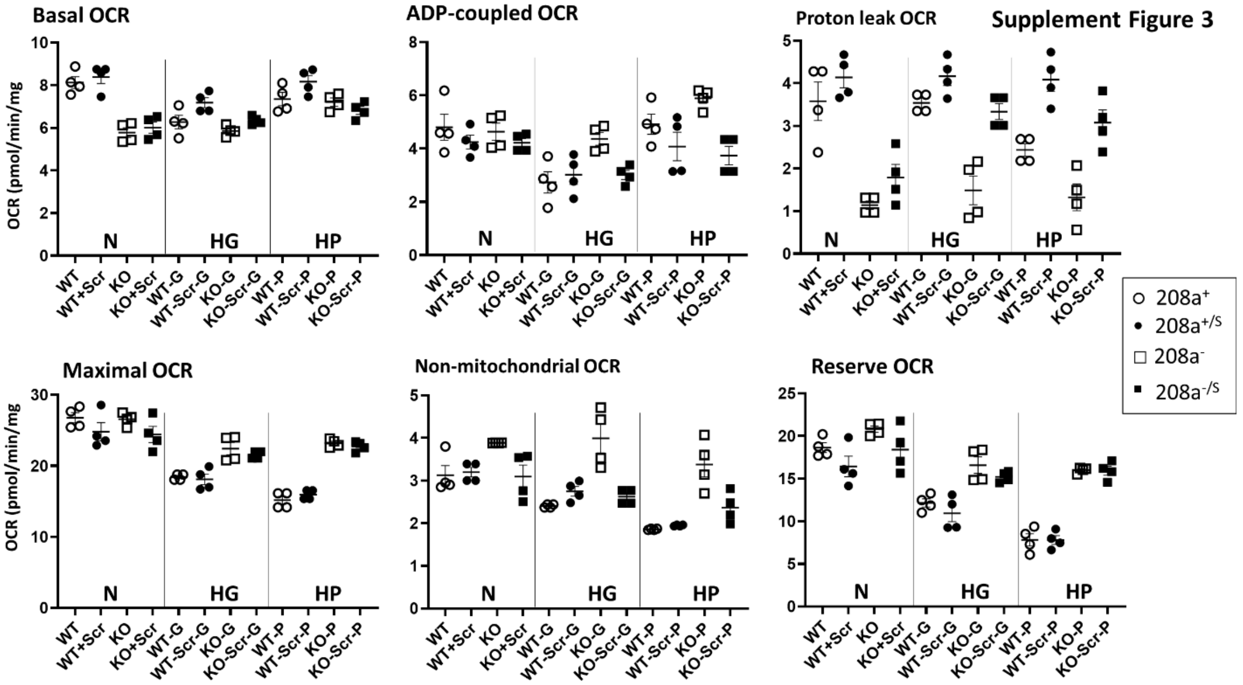

Supplementary Figure S3 (in support of Figure 5 in the main manuscript). The effect the scrambled control miR sequence on SV40 cardiomyocytes respiratory properties

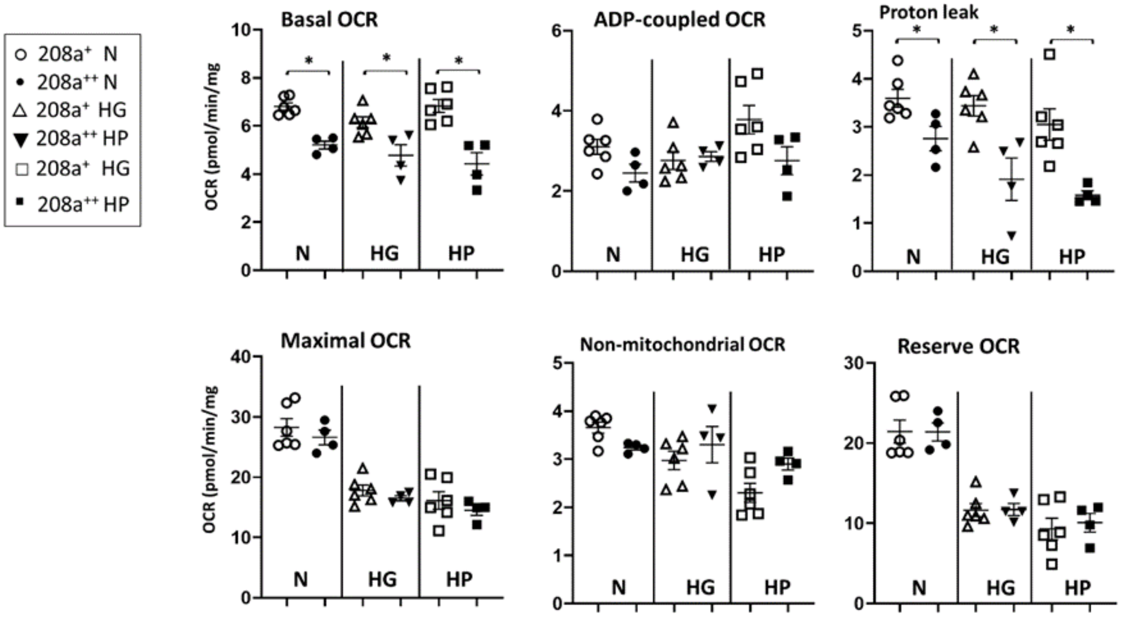

Supplementary Figure S4 (in support of Figure 5 in the main manuscript). A full description of the effect of miR 208 overexpression on mitochondrial respiratory properties

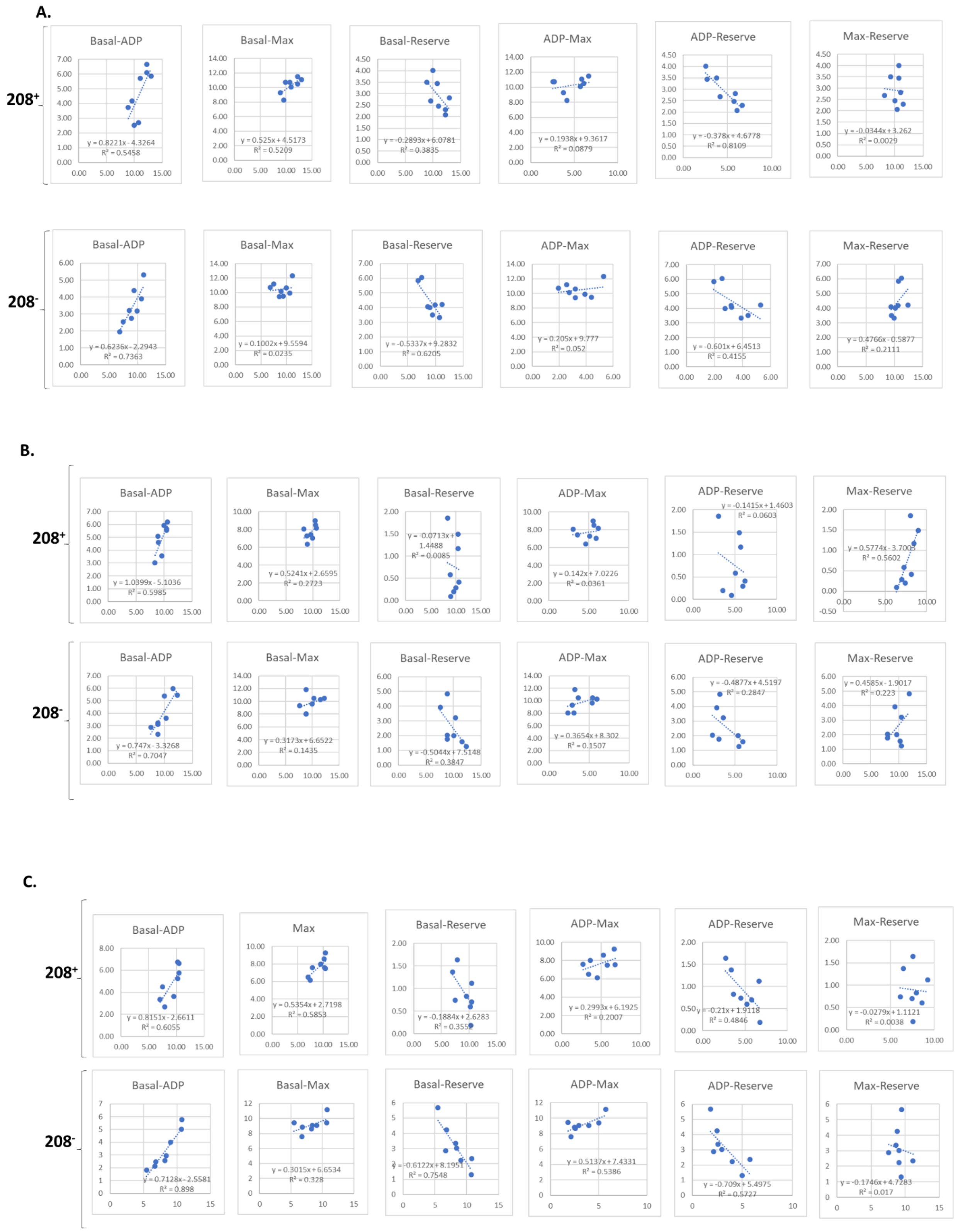

Supplementary Figures S5 (in support of Figure 5). Correlations between OCR parameters in human SV40 cardiomyocytes.
